# Targeting the Renin–angiotensin–aldosterone System (RAAS) for Cardiovascular Protection and Enhanced Oncological Outcomes: Review

**DOI:** 10.1007/s11864-024-01270-9

**Published:** 2024-10-18

**Authors:** J. Pawlonka, B. Buchalska, K. Buczma, H. Borzuta, K. Kamińska, A. Cudnoch-Jędrzejewska

**Affiliations:** Department of Experimental and Clinical Physiology, Laboratory of Centre for Preclinical Research, Medical University of Warsaw, Warsaw, Poland

**Keywords:** Cardiovascular Toxicity, Renin–angiotensin–aldosterone System

## Abstract

The renin–angiotensin–aldosterone system (RAAS) is a crucial regulator of the cardiovascular system and a target for widely used therapeutic drugs. Dysregulation of RAAS, implicated in prevalent diseases like hypertension and heart failure, has recently gained attention in oncological contexts due to its role in tumor biology and cardiovascular toxicities (CVTs). Thus, RAAS inhibitors (RAASi) may be used as potential supplementary therapies in cancer treatment and CVT prevention. Oncological treatments have evolved significantly, impacting patient survival and safety profiles. However, they pose cardiovascular risks, necessitating strategies for mitigating adverse effects. The main drug classes used in oncology include anthracyclines, anti-HER2 therapies, immune checkpoint inhibitors (ICIs), and vascular endothelial growth factor (VEGF) signaling pathway inhibitors (VSPI). While effective against cancer, these drugs induce varying CVTs. RAASi adjunctive therapy shows promise in enhancing clinical outcomes and protecting the cardiovascular system. Understanding RAAS involvement in cancer and CVT can inform personalized treatment approaches and improve patient care.

## Introduction

**Fig. 1:**
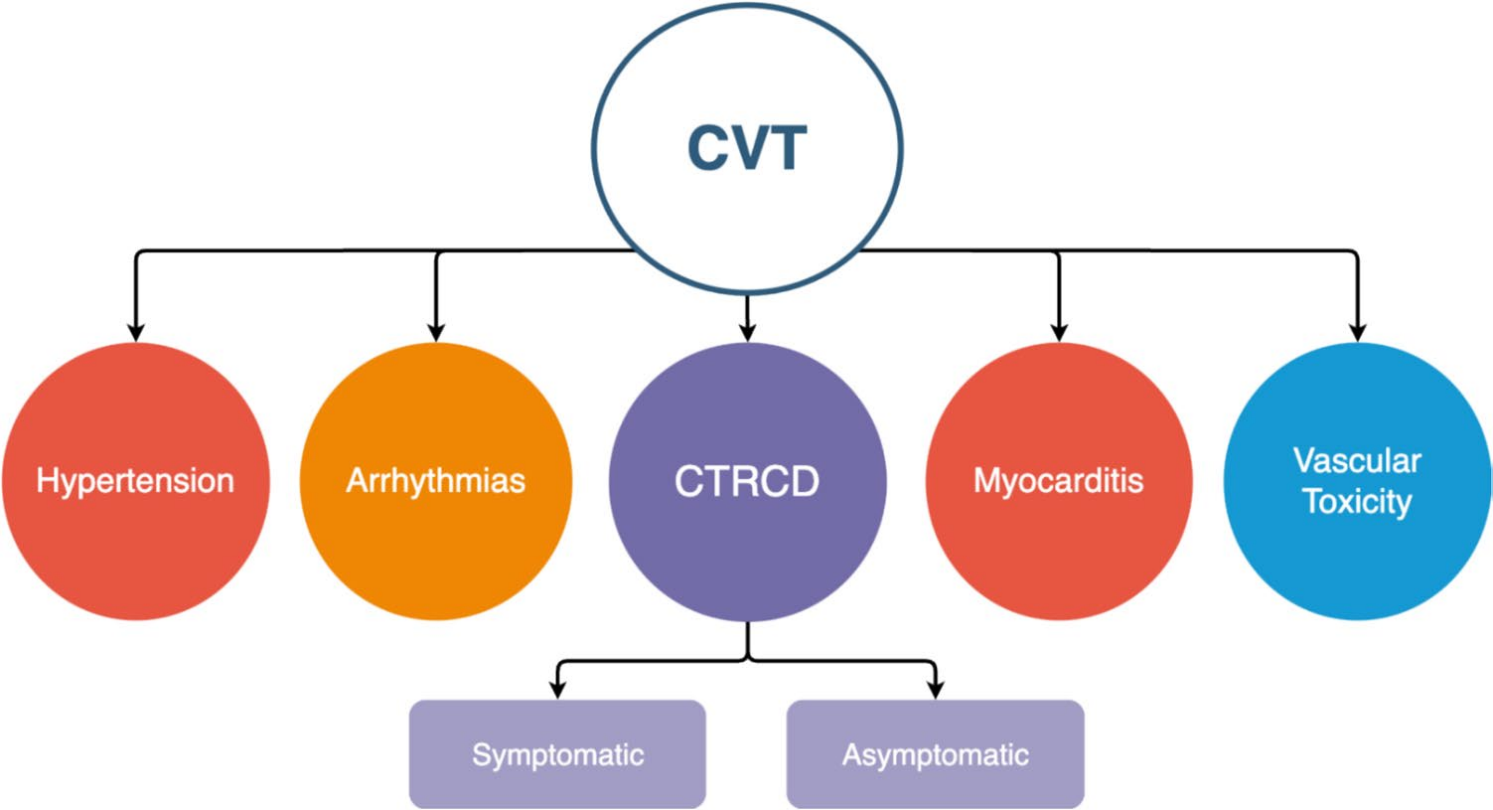
Definitions of cardiovascular toxicity (CVT). International Cardio-Oncology Society (IC-OS) consensus statement, in 2021, established standardized definitions of CVT, delineating five focus areas, and facilitating cross-disciplinary communication in both clinical practice and research. Herein, key classifications are shown. CTRCD—cancer therapy-related cardiac dysfunction.

Renin–angiotensin–aldosterone system (RAAS) serves as a key regulator of the cardiovascular system and is a target for widely used therapeutic drugs. Operating through two antagonistic axes – the classical and alternative (protective) axis – RAAS is physiologically balanced [1]. The dysregulation of this balance holds significance in the development of prevalent diseases such as hypertension and heart failure (HF). Recently, it has garnered attention in the context of oncological patients, where its abrupt activation has been demonstrated to play a crucial role in tumor biology [2, 3] and the development of cardiovascular toxicities (CVT) – see Fig. 1 [4–6]. Consequently, well-established RAAS inhibitors (RAASi), including angiotensin-converting enzyme (ACE) inhibitors (ACEIs), angiotensin receptor blockers (ARBs commonly referred to as sartans) and beta-blockers (BBs) have been suggested as potential supplementary therapies for cancer and/or agents for CVT prevention [7].

Oncological treatment schemes have dynamically evolved in recent years [8]. Targeted drugs and immunotherapy have significantly altered either overall survival (OS) or safety profiles for patients with prevalent oncological, hematological, and autoimmune diseases [9]. For patients diagnosed with metastatic breast cancer (BC) between 2008 and 2010 median OS was 31.1 months whilst for those diagnosed in 2017–2019 median OS was 38.4 months [10]. Similarly, patients with metastatic non-small cell lung cancer (NSCLC) treated with immunotherapy have higher median OS of 16.4 months than patients with NSCLC treated with chemotherapy (11.6 months) [9]. Alongside older, well-established chemotherapies, novel drugs constitute commonly used therapeutic schemes. Due to the steady decline in cancer-related mortality and the increasing number of cancer survivors, cardiovascular complications have gained new significance, as they have profound implications for prognosis. The discussion on how to increase the effectiveness of these therapies and reduce the risk of severe adverse effects, especially chronic ones, continues [7].

In our review we focus on four distinct classes of pharmaceutical drugs used in oncological treatment: (1) anthracyclines (e.g., doxorubicin) [11] (2) anti-HER2 (human epidermal growth factor receptor 2) therapies (e.g., monoclonal antibody trastuzumab) [12, 13], (3) immune-check-point-inhibitors (ICIs) (e.g., monoclonal antibodies: pembrolizumab, atezolizumab, ipilimumab) [14], (4) vascular endothelial growth factor (VEGF) signaling pathway inhibitors (VSPI) (e.g., monoclonal antibody bevacizumab [15], and tyrosine kinase inhibitors eg. sunitinib [16]) – see Table 1. These classes of drugs share common features. First and foremost, all the drug classes have been shown to cause CVT [13–17]. However, the profile of cardiotoxicity may differ significantly between the classes [18]. For instance, anthracyclines and anti-HER2 therapies mainly cause cardiac dysfunction, leading to symptomatic HF or asymptomatic reduction of left ventricle ejection fraction (LVEF) [17]. ICIs use is associated with severe myocarditis as well as an increased risk of major cardiac events, including myocardial infarction [14]. Finally, VSPIs mainly cause hypertension, thromboembolic events, or less commonly cardiac dysfunction [19]. All the drug classes are widely used in current therapeutic schemes for patients diagnosed with prevalent cancer types, including breast cancer (BC) [20, 21], lung [22, 23], gastrointestinal [24–26], and urogenital cancers [27, 28] as well as hematological malignancies, including Hodgkin (HL) [29] and non-Hodgkin lymphomas (NHL) [30, 31]. In some schemes, the drugs are used concomitantly [32, 33]. Ultimately, there is some evidence that the use of RAASi, concomitantly with these drugs, can improve clinical effectiveness and protect the cardiovascular system, potentially enhancing clinically relevant outcomes such as increased OS, progression free survival (PFS) or malignancy response rate (MRR) [33–37].

**Table 1:** Examples of clinical use of oncological drugs discussed in the review, based on European Society for Medical Oncology (ESMO) Guidelines

Oncological drugs		Example of clinical use			References
Class	Member	Cancer type	Stage	Therapy type	
Anthracyclines	Doxorubicin	HR + , HER- BC	early	(Neo)adjuvant	Loibl et al. [20]
		TN BC	early	(Neo)adjuvant	Loibl et al. [20]
		TN BC	metastatic	Palliative	Gennari et al. [21]
		HL	Limited Intermediate Advanced	part of ABVD, AVD, BEACOPPes	Eichenauer et al. [29]
		FL	high tumor burden, aggresive,	part of R-CHOP	Dreyling et al. [30]
		DLBCL	According to aaIPI and maximum bulk of disease	part of ACVBP and (R)-CHOP	Tilly et al. [31]
		MCL	stages III-IV*	part of R-CHOP and VR-CAP	Dreyling et al. [30]
	Epirubicin	TN BC	metastatic	Palliative	Gennari et al.[21]
Anti-HER2 agents	Trastuzumab	HER2 + BC	early	(Neo)adjuvant	Loibl et al. [20]
		HER2 + BC	metastatic	Palliative	Gennari et al. [21]
		HER2 + GC	metastatic	Palliative	Lordick et al. [24]
	Pertuzumab	HER2 + BC	metastatic	palliative	Gennari et al. [21]
VSPI	Bevacizumab	RCC	metastatic	Palliative	Escudier et al. [28]
		CRC	metastatic	Palliative	Cervantes et al. [26]
		Luminal A or B BC	metastatic	palliative	Gennari et al. [21]
		HER2- BC	metastatic	Palliative	Gennari et al. [21]
		TN BC	metastatic	Palliative	Gennari et al. [21]
	Sunitinib	RCC	metastatic	Palliative	Escudier et al. [28]
	Sorafenib	HCC	metastatic	Palliative	Vogel et al. [25]
	Pazopanib	RCC	metastatic	Palliative	Escudier et al. [38]
	Cabozantinib	RCC	metastatic	Palliative	Escudier et al. [38]
		HCC	metastatic	Palliative	Vogel et al. [25]
ICI	Pembrolizumab	NSCLC	metastatic	Palliative	Hendriks et al. [23]
		HCC	metastatic	Palliative	Vogel et al. [25]
		UC	metastatic	Palliative	Powles et al. [27]
		GC	metastatic	Palliative	Lordick et al. [24]
		CRC	metastatic	Palliative	Cervantes et al. [26]
	Nivolumab	NSCLC	metastatic	Palliative	Hendriks et al. [23]
		HCC	metastatic	Palliative	Vogel et al. [25]
		PD-L1 + GC	metastatic	Palliative	Lordick et al. [24]
		RCC	metastatic	Palliative	Escudier et al. [28]
	Ipilimumab	NSCLC	metastatic	Palliative	Hendriks et al. [23]
		RCC	metastatic	Palliative	Escudier et al. [28]
	Atezolizumab	NSCLC	metastatic	Palliative	Hendriks et al. [23]
		SCLC	metastatic	Palliative	Dingemans et al. [39]
		UC	metastatic	Palliative	Powles et al. [27]
		TN BC	metastatic	Palliative	Gennari et al. [21]
	Durvalumab	NSCLC	metastatic	Palliative	Hendriks et al. [23]
		SCLC	metastatic	Palliative	Dingemans et al. [39]
		TN BC	early	(Neo)adjuvant	Loibl et al. [20]

*aa(IPI)* age-adjusted international prognostic index, *ABVD* doxorubicin/bleomycin/vinblastine/dacarbazine, *AVD* doxorubicin/ vinblastine/ dacarbazine, *BEACOPPesc* bleomycin/ etoposide/ doxorubicin/ cyclophosphamide/vincristine/procarbazine/prednisone chemotherapy, *BC* breast cancer, *CRC* colorectal cancer, *DLBCL* diffuse large B-cell lymphoma, *GC* gastric cancer, *HER2*, *HL* Hodgkin lymphoma, *HR* hormone receptor, *ICI* immune check-points inhibitor, *MCL* mantle cell lymphoma, *NSCLC* non-small-cell lung carcinoma, *PD-L1* programmed cell death protein-1 ligand, *RCC* renal cell carcinoma, *R-CHOP* rituximab cyclophosphamide, doxorubicin, vincristine and prednisone, *TN* triple negative, *UC* urothelial carcinoma (bladder cancer), *VR-CAP* rituximab, cyclophosphamide, doxorubicin and prednisone with bortezomib. *Lugano classification

Such effects have been extensively studied in preclinical in vivo trials [6, 40, 41]. RAAS has been shown to modulate heart muscle tissue, coronary circulation and arterial blood pressure. The classical RAAS axis promotes maladaptive ventricular remodeling and cardiac dysfunction, while the protective axis counterbalances the effect [1, 6, 40]. During cancer treatment the axes are dysregulated, leading to the increased cardiovascular risk [2]. Simultaneously, RAAS components are dysregulated in tumor microenvironment [3, 42], typically leading to the overexpression of classical axis compounds [43, 44]. RAAS dysregulation affects cancer cells and the surrounding stroma, vasculature, and local immune cells, promoting angiogenesis [45]. It may also influence invasion, prosurvival signaling, and proliferation, which contributes to the development of malignancy [2].

In our paper, we provide a comprehensive overview of the role of RAAS in CVT and malignancy development. We place particular emphasis on the application of RAASi in clinical practice as both a preventive strategy for CVT and a supplementary therapy that has the potential to enhance the efficacy of oncological treatment. Additionally, we explore reports suggesting that RAASi may influence the increased incidence of some cancers, such as lung cancer, negatively impact the tumor microenvironment, and diminish the effectiveness of chemotherapy.

## Renin–angiotensin–aldosterone system (RAAS)

**Fig. 2:**
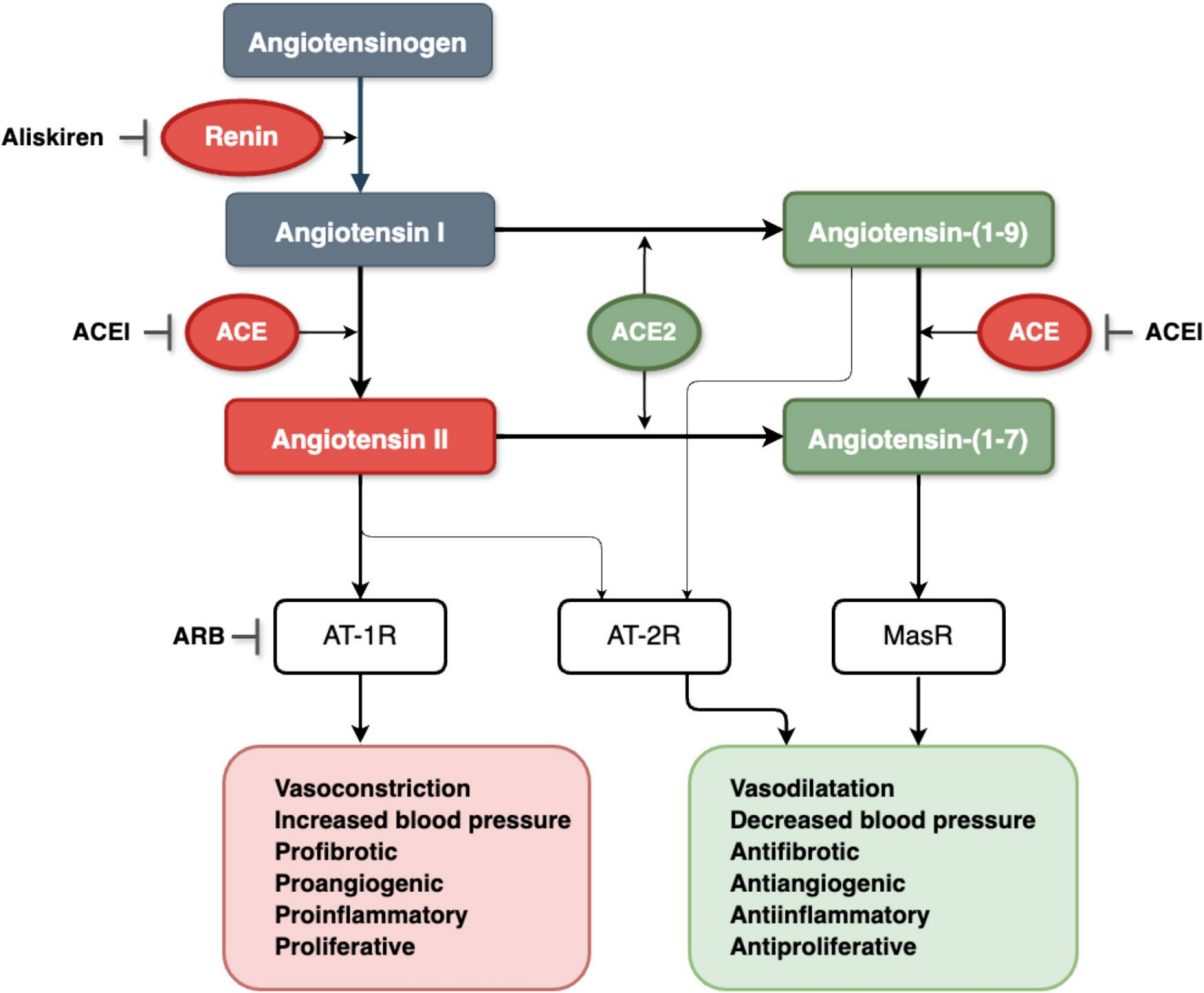
Schematic presentation of renin–angiotensin–aldosterone system (RAAS) and effects mediated by main angiotensin receptors. ACE – angiotensin-converting enzyme, ACE2- angiotensin-converting enzyme type 2, ACEI – angiotensin-converting enzyme inhibitor, ARB – angiotensin II receptor blocker, AT-1R—angiotensin receptor type 1, AT-2R angiotensin receptor type 2, Mas1R—G-protein-coupled receptor Mas.

RAAS appears deceptively simple as a homeostatic system, long recognized as a crucial determinant of arterial blood pressure, electrolyte and fluid balance – see Fig. 2 [46]. However, in reality, it constitutes a comprehensive and intricate enzymatic and hormonal cascade, operating through various receptors and signaling assemblies [2]. Beyond its systemic functions, it has been shown to modulate inflammation, fibrosis, cell proliferation, angiogenesis, and apoptosis at the tissue level [47, 48]. Additionally, the cloning of genes for the main RAAS components has revealed their expression in unexpected tissues other than liver (angiotensinogen), kidney (renin) and lung (ACE). The components and their receptors have been identified in various organs, including brain, blood vessels, and heart [48], as well as in numerous cancer tissues and cell lines, such as BC [49], ovarian [43], prostate [44], pancreatic [50] and gastric cancer [51]. Therefore, dysregulation of RAAS may be involved not only in pathophysiology of common diseases like hypertension, HF or nephropathy but also in the development of malignancy [49, 50] or in toxicities induced due to the oncological treatment, particularly CVT [4].

RAAS compounds are organized into two main axes: the classical or conventional axis involves ACE, angiotensin II (Ang-II), and angiotensin receptor type 1 (AT-1R) and is called ACE/Ang-II/AT-1R axis. The alternative involves ACE2, angiotensin-(1–7) (Ang-(1–7)), and MasR (G-protein-coupled receptor Mas)—ACE2/ang-(1–7)/MasR axis [1]. These axes exert opposing effects [52]. The classical axis mediates well-recognized cardiovascular actions, with AT-1R activation by Ang-II leading to vasoconstriction, water intake, Na^+^ retention, and consequent blood pressure elevation. Its extended roles include the induction of adverse tissue remodeling due to inflammation, oxidative stress, hypertrophy, and fibrosis [47]. In contrast, the ACE2/ang-(1–7)/MasR axis counteracts the activities of the classical axis [52]. Their effects include vasodilatation, decreased blood pressure, improved renal blood flow, as well as tissue-protective actions such as anti-inflammation, anti-fibrosis and anti-apoptosis [53, 54]. Dysregulation or imbalance between the classical and alternative axes may underpin several cardiovascular or oncological diseases [2].

Systemic RAAS activation is triggered by renin, released by the juxtaglomerular apparatus of the kidney. Renin release can be a response to decreased blood pressure in the renal arterioles, reduced blood volume, low sodium levels in the kidney tubules, or the excitation of b1-adrenergic receptors [55]. Renin as a proteinase, cleaves angiotensinogen (ATG) – an alpha-2-globulin– into Ang-I [56]. Ang-I is an inactive peptide but serves as the precursor for significant signaling molecules: Ang-II and angiotensin-(1–9) (Ang-(1–9)) [54]. The conversion of Ang-I to Ang-II is facilitated by ACE, primarily found on the surface of endothelial cells throughout the body, with abundant presence in the lungs, intestine and on brush border membranes in the kidney [57]. Ang-II, the central and most potent RAAS effector molecule, exerts a wide range of physiological effects by binding G-protein-coupled receptors: AT-1R—the principal RAAS receptor or to a lesser extent AT-2R, which is critical mainly in the prenatal period [1]. The effects of the conventional RAAS axis are mediated by Ang-II-AT-1R binding, with elevated Ang-II tissue levels observed in various diseases, including hypertension and/or HF [48]. The Ang-II-AT-2R receptor binding usually has contrary effects, playing a protective role, consistent with other AT-2R ligands such as angiotensin III (Ang-III) and Ang-(1–9) considered protective as well [1].

The main component of the classical RAAS axis, Ang-II, can be alternatively modified by other enzymes (aminopeptidase A and aminopeptidase N), producing Ang-III and angiotensin IV (Ang-IV) respectively [58]. These products may interact with different specific receptors, with Ang III acting through AT-1R and AT-2R receptors, and Ang-IV through its specific receptor AT-4R (known as insulin-regulated amino peptidase—IRAP) [59].

The discovery of the components of the ACE2/ang-(1–7)/MasR axis has redefined the form and function of RAAS [1, 54]. ACE2, a carboxypeptidase, is primarily expressed on the surface of certain endothelial cell populations, with the most abundant expression in the kidneys, followed by the heart. ACE2 cleaves Ang-I to Ang-(1–9), which can be further converted by ACE to Ang-(1–7) [1]. The conversion of Ang-I to Ang-(1–7) may be also direct (Santos et al.[1]). Ang-(1–7) binds to MasR, however, it can slightly exert its effects through AT-2R or MAS1-related G-protein-coupled receptor D (MrgD) binding [1]. The main conversions of the RAAS peptides, receptor binding and effects are shown in Fig. 2.

The ultimate compound of RAAS is aldosterone, a steroid hormone produced by the adrenal cortex and released in response to Ang-II [60]. The mineralocorticoid receptor (MR) specific to aldosterone is mainly found in the kidneys, where its excitation promotes the secretion of potassium ions and secondary the retention of sodium ions and water, contributing to the regulation of fluid volume and blood pressure [60].

## Renin–angiotensin–aldosterone system inhibitors (RAASi)

Given the physiological significance of RAAS and its involvement in prevalent cardiovascular and renal diseases, specific targeting drugs have been developed. These drugs, known as RAASi, encompass ACEIs, ARBs, angiotensin receptor/neprilysin inhibitors (ARNIs), mineralocorticoid receptor antagonists (MRA), known as aldosterone antagonists, and direct renin inhibitors [61, 62]. ACEIs and ARBs, particularly, form the cornerstone of effective treatments for conditions like hypertension, HF, and kidney diseases [61, 63]. Recently, there has been a growing exploration of their potential role in preventing and treating CVT and renal toxicity [64, 65].

ACEIs competitively target ACE, a dipeptidyl carboxypeptidase A, which cleaves dipeptides from the C-terminus of various proteins like Ang-I, bradykinin, enkephalins, proenkephalins, substance P, luteinizing hormone releasing hormone [66, 67]. ACE is mainly bound to the plasma membranes and exists in three types – endothelial, epithelial, and neuroepithelial – each with distinct roles in physiological processes [57]. ACE consists of two homologous domains – nACE and cACE with a similar structure but distinctive substrate specificities [68]. The cACE influences mainly blood pressure whilst nACE controls the proliferation of hematopoietic stem cells [69].

ACEIs are classified into three groups, according to the chemical structure: (1) Sulfhydryl-containing ACE inhibitor – captopril, (2) Phosphorus-containing ACE inhibitor – Fosinopril and (3) Dicarboxylic-containing ACE inhibitors such as enalapril, lisinopril, ramipril and perindopril [70]. Almost all the ACEIs are prodrugs activated in the liver, except for lisinopril and captopril. Almost all are administrated orally, except for enalapril which can be administrated intravenously as well [70]. The majority of selective ACEIs bind to non-conserved amino acids between the two domains nACE and cACE [69]. Both ACE domains have similar structure – they are ellipsoid and contain central Zn2 + ion. Around the ion there are four subsites in each domain – S2, S1, S1’, and S2’ [68]. It was found that lisinopril interacts with ACE adjacently to the HExxH motif and catalytic Zn2 + ion. The phenyl ring of lisinopril binds with S1 subsite in active center, lysine of lisinopril binds with S1’ subsite, and proline—with S2’ subsite. The carboxyl group of lisinopril binds to Zn2 + and has H-bond with Glu384 [71]. Captopril as a smaller compound lacks interactions with S1 subsite. This drug binds Zn2 + together with Glu338 through a sulfhydryl group not a carboxyl group [71]. ACEIs have different inhibitory potencies to nACE and cACE. Lisinopril has higher inhibitory potency for cACE than enalapril and captopril, but captopril has higher inhibitory potency for nACE than lisinopril and enalapril [72]. While currently existing ACEIs target both nACE and cACE Alves-Lopes et al. have shown selective inhibition of cACE may lower Ang-II concentration without increasing bradykinin levels thus preventing side effects [73]. Captopril, unlike other ACEIs, has short half-life of 0.81 h after acute and 0.96 h after chronic administration [74, 75]. Other ACEIs like enalapril and lisinopril have half-lives of about 10 h [74].

Because of a lack of ACEIs selectivity, and interactions with both ACE domains, many side effects may arise [68]. In the study on rat pulmonary ACE Perich et al. reported there are differences in binding affinity of different ACEIs to the two binding domains. A decrease in binding affinity was associated with an increase in ACEI side chain length and complexity of C-terminal moiety [76]. Compared with ARBs, ACEIs inhibit effect of Ang-II on both AT-1R and AT-2R [77].

ARBs inhibit only AT-1R and block the effects of a peptide agonist Ang-II [78]. This results in a two- to threefold rise in circulating Ang-II levels, which may bind to AT-2Rs [79]. ARBs treatment is also associated with increased concentrations of renin and Ang-I [77]. The non-peptide ARBs (sartans) bind between transmembrane regions. When these antagonists have carboxyl groups, they interact with the protonated amino group of the Lys199 in the fifth transmembrane helix. Losartan binds to amino acids located in the second to seventh transmembrane helices. The extracellular residues of AT-1R play important role in Ang-II binding but not in receptor activation [80]. Losartan-receptor complex is not internalized and does not have impact on cell surface receptors population [79]. The non-peptide ARBs can be divided into insurmountable (binding to transmembrane helices of AT-1R changes conformation of the receptor preventing ang-II binding) and more common competitive (Ang-II interacts with the same part of receptor as ARB) [80].

ACEIs serve cardioprotective functions mainly through blockade of conversion of Ang-I to Ang-II, kinin hydrolysis, and stimulation of prostaglandin production [81, 82]. Increased bradykinin levels promote nitric oxide production and thus lead to cardioprotection [83]. The sulfhydryl-group-containing ACEIs may have additional beneficial effects due to interactions with bradykinin and scavenging free radicals [82]. Apart from that, Peng et al. have shown inhibiting hydrolysis of N-acetyl-seryl-aspartyl-lysyl-proline (Ac-SDKP) in rats may also have cardiac antifibrotic action. ACEIs decreased left ventricular collagen along with monocyte/macrophage infiltration, cell proliferation, and transforming growth factor beta1 TGF-beta expression [81]. ARBs on the other hand lack the cardioprotective effects [84]. Such difference has been confirmed in the clinical setting. In a systematic review of eight RCTs in 37,148 patients with ischemic heart disease, ACEIs administration was associated with a reduced risk of total mortality (RR 0.87, 95% CI 0.81–0.94) and cardiovascular mortality (RR 0.83, 95% CI 0.70–0.98) compared with placebo, whilst these risks remained unchanged with ARBs.[85]. In a meta-analysis of 26 RCTs in 108,212 high cardiovascular-risk patients without HF, ACEIs significantly reduced the risk of all-cause death (OR 0.908, 95% CI 0.845–0.975; p = 0.008), MI (OR 0.811, 95% CI 0.748–0.879), and new-onset HF (OR 0.789, 95% CI 0.686–0.908), whilst there was no significant reduction of these risks with ARBs (all-cause death OR 1.006, 95% CI 0.941–1.075; p = 0.866; MI OR 0.903, 95% CI 0.803–1.015; p = 0.086; and new-onset HF OR 0.892, 95% CI 0.761–1.046; p = 0.159) [86, 87]).

On the other hand, the use of ARBs was associated with lower risk of adverse effects, including angioedema, cough, pancreatitis, gastrointestinal bleeding, and abnormal weight loss [88]. Li et al. in a systematic review reported patients treated with ACEIs and ARBs have similar risk of death due to heart disease, or total heart disease and stroke [89]. However, ARBs have 1.8% lower chance of being stopped as a result of adverse effects in 4.1-years period. This is mainly because ACEIs cause dry cough in more patients [89]. The cause of lower rate of side effects during ARBs treatment may be they act further downstream the angiotensin cascade than ACEIs [90].

ARNIs, a class of drugs, with their first member sacubitril/valsartan, arose after positive conclusions from PARADIGM-HF trial, which evidenced their particular efficacy in patients with HF with reduced ejection fraction [62]. Sacubitril/valsartan inhibits both angiotensin receptors and neprilysin and it is more efficient than enalapril in decreasing the risk of worsening of HF, the need for more intensive treatment, or heart transplantation in patients with HF with reduced ejection fraction (HFrEF) [91]. The cardioprotective effects of ARNIs are associated with decreased oxidative stress levels, the inhibition of myocardial inflammatory response, protection against mitochondrial damage and endothelial dysfunction, and improvement in the RAAS imbalance [92]. The most frequent adverse effect of sacubitril/valsartan is hypotension, and the risk is higher than for ACEI or ARB [93]. Compared to ACEIs and ARBs sacubitril/valsartan reduces the risk of new-onset diabetes mellitus more, however, it is associated with higher risk of hypoglycemia [94].

MRAs inhibit the actions of aldosterone and are used in the management of both hypertension and HF. This class of drugs includes spironolactone, eplerenone, and canrenone [95]. MRAs are known to reduce extracellular matrix turnover and myocardial collagen levels as well as improve endothelial vasomotor dysfunction, protecting the heart muscle [96]. Some animal models have tested eplerenone in the context of anthracycline-induced cardiotoxicity, leading to inconsistent results [97, 98].

## The interplay between RAAS and CVT: lessons from animal models of oncological treatment

RAAS affects the heart both directly by modulating heart muscle tissue and indirectly through changes in coronary vasculature and arterial blood pressure [47]. RAAS compounds are derived not only from the plasma but are also locally generated in tissues from precursors that are either expressed locally or imported from the circulation [48]. The classical ACE/ang-II/AT-1R axis promotes oxidative stress, hypertrophy, fibrosis, and inflammation in the myocardium, contributing to maladaptive ventricular remodeling and organ dysfunction [47]. ACE2 and Mas are expressed in cardiomyocytes, cardiofibroblasts, and the coronary vasculature to counterbalance Ang-II signaling [1].

Certain oncological drugs alter the activity of RAAS components, with the most extensive data available on AIC. In preclinical animal studies, doxorubicin has been shown to disrupt the balance between the classical RAAS axis and its protective antagonist. Doxorubicin administration triggered a significant increase in Ang-II levels in plasma of male Sprague–Dawley rats [99]. The drug was given over a period of 2 weeks with a cumulative dose of 15 mg/kg [99]. Doxorubicin also increased Ang-II levels in the myocardium and in cardiovascular regulation centers of male Sprague–Dawley rats when given at 3 mg/kg/day for 12 days [100]. Increased ACE activity induced by doxorubicin has been observed in the myocardium and kidneys of male Syrian hamsters [101]. Doxorubicin was given as a single intravenous injection in a dose of 1.5 mg/kg [101]. The baseline ACE activity may correlate with kidney damage as was shown on male Wistar rats which received a single intravenous injection of doxorubicin 1.5 mg/kg [102]. Doxorubicin causes AT-1R overexpression and concomitant downregulation of the protective receptor AT-2R as was shown in male Sprague–Dawley rats (6 intraperitoneal injections and a cumulative dose of 15 mg/kg) [6]. Furthermore, *AGTR1* knock-out mice (AT1KO mice) treated with doxorubicin (a single injection of 20 mg/kg dose and then 1 mg/kg once a week for 12 weeks) did not develop maladaptive structural changes in cardiomyocytes or macroscopic structural (left ventricle dimensions) and functional (LVEF) changes [40]. Finally, doxorubicin lowers Ang-(1–7) levels and may reduce myocardial MasR expression in mice (a single 10 mg/kg dose of doxorubicin), deepening the imbalance between an overactivated classical axis and a suppressed protective axis [5].

Dysregulation of RAAS activity has also been demonstrated in VSPI-induced CVT, where hypertension and HF are the main complications [103, 104]. In male mice receiving bevacizumab (10 mg/kg intravenously for 4 weeks) and sunitinib (40 mg/kg orally for 4 weeks), RAASi prevented the development of hypertension and partially attenuated adverse cardiovascular remodelling and cardiac dysfunction, suggesting an important role of RAAS signaling in the development of cardiotoxicity [41]. The study proposed that the cardioprotective effects of RAAS antagonists were not entirely dependent on their blood pressure-lowering capabilities [41]. In male Wistar Kyoto rats receiving sunitinib (26.7 mg/kg per day for 8 days) and captopril (3 to 12 mg/kg), didn’t prevent a rise in arterial blood pressure but was effective in preventing proteinuria, suggesting its nephroprotective efficacy [105]. However, these studies didn’t specify the changes in specific RAAS compounds and were not sufficient to assess whether the changes in RAAS activity were systemic or local [41, 105].

## The Interplay between RAAS and tumor microenvironment: impact on tumor progression and treatment strategies

In oncological patients RAAS has been demonstrated to play a significant role not only in drug-induced organ toxicities but also in tumor biology [2]. Components of the RAAS are dysregulated in human malignancies and inhibition of RAAS may correlate with disease outcomes [3, 42]. In a retrospective cohort study involving 5207 hypertensive patients between 1980 and 1995, it was found that the use of angiotensin-converting enzyme (ACE) inhibitors was significantly associated with a reduced risk of cancer [3]. Patients receiving ACE inhibitors had a relative risk of incident cancer of 0.72 (95% CI 0.55–0.92) and a relative risk of fatal cancer of 0.65 (0.44–0.93) compared to the control group [3]. Furthermore, among women, the use of ACE inhibitors was associated with the lowest relative risk of cancer, including female-specific cancers [3]. Moreover, the reduced relative risk of cancer in patients on ACEI was greatest with follow-up of longer than 3 years, suggesting potential benefits of long-term use of these drugs [3]. In another retrospective analysis of 287 patients with advanced NSCLC undergoing first-line platinum-based chemotherapy, it was found that those receiving ACEI or ARB had a median survival 3.1 months longer than non-recipients (11.7 vs. 8.6 months, HR 0.56, P = 0.03), highlighting the potential of these drugs as adjunctive therapy in oncological settings [42].

The effect is primarily mediated by abrupt alteration of localized organ RAAS mechanisms [106]. During tumorigenesis the expression of certain components undergoes changes, typically leading the overexpression of classical axis compounds, including AT-1R and ACE [43, 44].

Suganuma et al. investigated in vitro the expression of AT-1R in human ovarian carcinoma. In a cohort of ovarian tumor tissues (n = 99), AT-1R expression was found in the majority of invasive carcinomas (57 out of 67; 85%) and borderline malignant tumors (12 out of 18; 66%), but only minimally in benign cystadenomas (2 out of 14, 14%) [43]. Among invasive carcinomas, cases strongly positive for AT-1R (n = 37) exhibited significantly higher levels of VEGF expression and intratumor microvessel density compared to cases weakly positive (n = 20) or negative (n = 10) for AT-1R [43]. In vitro experiments using SKOV-3 ovarian cancer cells demonstrated that Ang-II enhanced invasive potential and VEGF secretion, both of which were effectively inhibited by the AT-1R blocker candesartan [43]. Furthermore, in a mouse model of SKOV-3 transplanted peritoneal carcinomatosis, administration of candesartan led to reduced peritoneal dissemination, decreased ascitic VEGF concentration, and suppression of tumor angiogenesis [43]. Uemura et al. studied in vitro the expression of RAAS components in hormone refractory prostate cancer (HRPC) in 87 prostate tissue samples. The expression of AT-1R, ACE and Ang-I/II precursor was significantly higher in HRPC than that in normal prostate tissue and untreated prostate cancer tissue [44]. The study showed that Lymph Node Carcinoma of the Prostate (LNCaP) cells, when stimulated with dihydrotestosterone (DHT), estradiol (E2), dexamethasone (DEX) or anti-androgen drugs, have increased protein expression of the AT-1R and Ang-I/II in vitro, suggesting the expression of RAAS components may be altered by certain kinds of hormonal stimulation [44]. Moreover, the expression of these components correlates with specific stages in cancer progression and may vary between in situ lesions and invasive cancers [107]. De Paepe et al. in an in vitro study (controls (n = 10), patients with hyperplasia (n = 33), ductal carcinoma in situ (DCIS) (n = 23) and invasive carcinoma of the breast (n = 25)) showed specific overexpression of AT-1R on the cytoplasmic membrane of cells of hyperplastic lesions with and without atypia and on DCIS of the breast [107]. Interestingly, the expression of AT-1R on the cell membrane diminished in invasive breast cancer cells, indicating a regulatory pathway no longer required in invasive carcinoma [107]. In vitro experiments demonstrated growth induction by angiotensin II in breast-derived T-47D cells expressing AT-1R but not AT-2R, confirming a role in growth stimulation [107].

RAAS dysregulation affects either cancer cells or the surrounding stroma and vasculature, leading to increased VEGF expression and the promotion of angiogenesis as was shown on mice models [45]. Tumors introduced into the subcutaneous tissue of wild-type mice exhibited robust angiogenesis, characterized by the induction of VEGF within the tumor stroma, where the expression of the AT-1aR was detected (while AT-1bR and AT-2R expression was absent) [45]. Administration of a systemic AT-1R antagonist led to a decrease in tumor-associated angiogenesis and VEGF expression within the tumor stroma [45]. Furthermore, in AT-1aR null mice, compared with the wild-type, both tumor-associated angiogenesis and the expression of VEGF in the stroma were reduced, suggesting that host stromal VEGF induction by AT-1aR signaling is one of a key regulator of tumor-associated angiogenesis and tumor growth [45].

Alterations of RAAS components may result from several, possibly overlapping, mechanisms, such as germline and somatic mutations [49], the activation of upstream signaling pathways [50, 108] or microRNAs (miRNAs) [109]. However, the question of whether this dysregulation is causative, or a consequence of malignancy remains unresolved [2].

Dysregulation of the RAAS contributes to the development of hallmark capabilities during malignant transformation [110]. This impact is most pronounced in angiogenesis, invasion, prosurvival signaling and proliferation [2]. All these processes are interdependent and cooperative [110].

The use of RAASi use may counteract the abrupt RAAS activation in the tumor microenvironment. They have been shown to reduce tumor growth and/or progression in numerous cancers in both preclinical [111] and clinical trials [15, 112, 113]. Araujo et al. investigated the effects of RAAS blockade on RCC in a murine BALB/c model [111]. They injected murine renal cancer cells (Renca) into the subcapsular space of the left kidney and treated animals with losartan or captopril or both, administrated by gavage for 21 days after inoculation [111]. Animals subjected to treatment displayed diminished tumor sizes, irrespective of the treatment protocol, and exhibited significantly fewer lung metastases in terms of both number and size compared to the untreated group [111]. The levels of VEGF and CD34 expression were notably reduced in renal tumors of treated animals in comparison to the untreated counterparts [111]. Sun et al. performed a meta-analysis of 55 studies to evaluate the association between RAASi and recurrence, metastasis and survival in cancer patients [113]. They showed improvements in OS (HR = 0.82; 95% CI: 0.77–0.88; P < 0.001), PFS (HR = 0.74; 95% CI: 0.66–0.84; P < 0.001) and disease-free survival (HR = 0.80; 95% CI: 0.67–0.95; P = 0.01) in RAASi users compared with non-users [113]. Subgroup analyzes indicated a noteworthy enhancement in OS among individuals utilizing ARBs (HR = 0.80; 95% CI: 0.67–0.95; P = 0.01), whereas only a marginal enhancement in OS was observed among ACEI users (HR = 0.94; 95% CI: 0.85–1.04; P = 0.27) [113]. The results revealed a significantly better outcome in OS among RAASI users with certain tumor types, including renal cell carcinoma (RCC), gastric cancer (GC), pancreatic cancer (PC) and hepatocellular carcinoma (HCC) [113]. RAASi did not seem to influence OS in patients with esophageal carcinoma, breast cancer, and biliary tract cancer [113]. Finally, there were negative effects on OS in acute myelocytic leukemia and multiple myeloma in RAASI users compared with non-users [113].

At this point, we need to distinguish two kinds of studies: those showing the influence of RAASi on the risk of cancer development [114–116] and those demonstrating the benefits of RAASi administration in cancer patients at various stage [112]. Some retrospective analyzes also showed that RAASi administration increases OS in cancer patients [42, 113], however, some studies presented contrary results [117]. It must also be noted that one large-scale meta-analysis showed a modest but significant increase in the risk of developing cancer with the use of ARBs. However, among solid tumors only the risk of developing lung cancer was increased [118]. Other, recently published meta-analyses, showed inconsistent results whether ACEIs use was linked to increased lung cancer risk [115, 116]. Moreover, one showed that the risk of developing lung cancer was greater for ACEIs users than ARBs users [115].

## RAASi in anthracycline ± anti-HER2 agents cancer therapy: navigating cardioprotection, survival, and treatment response

Anthracyclines are firmly established as oncological drugs and are consistently utilized key components in chemotherapy for BC [20, 21] and hematological malignancies, including HL [29] and NHL [30, 31]. AIC has been the focal point of numerous studies, investigating mechanisms of toxicity, changes in endogenous hormonal axes, as well as the prevention and treatment strategies. Among the investigated mechanisms, dysregulation of RAAS has been identified as a significant factor [99, 100]. There is also substantial evidence supporting the use of RAAS antagonists in preventing AIC, with reliable foundations found in pre-clinical studies [6, 40].

In recent years, anti-HER2 regimens, including monoclonal antibody trastuzumab, have become integral to BC treatment protocols in both early and metastatic settings [20, 21]. However, it has been recognized that their utilization can lead to cardiotoxicity [13]. HER2-targeted therapies have also been demonstrated to exacerbate the cardiotoxic effects of anthracyclines [119]. We have decided to discuss these drugs in a single chapter alongside anthracyclines, as they are commonly used together in breast cancer treatment, and a significant portion of the research includes patients treated with both groups.

Several randomized controlled trials (RCTs) showed the positive impact of RAAS blockade in mitigating the decline of LVEF and reducing the incidence of HF. The data regarding the impact of RAASi on OS and PFS in cancer patients treated with anthracyclines and/or anti-HER2 drugs, however, is still not conclusive [33, 34, 120]. In many aspects, meta-analyzes and systematic reviews exhibit heterogeneity: (1) some exclusively incorporate RCTs, while others also encompass observational studies [121], (2) certain studies examine the combined effects of protective drugs from various classes, for example, ACEIs + beta-antagonists versus placebo [11, 12, 122], while others focus solely on specific protective drugs in monotherapy, such as enalapril (an ACEI) versus other ACEIs/ARBs versus placebo [123], (3) studies involve patients post-anthracycline [123], trastuzumab [12], or anthracycline + trastuzumab medication treatment [11, 122], (4) there is variation in sex ratios across studies, with some exclusively including women [12], (5) trials encompass different malignancies, with some concentrating solely on BC [11, 12] and others having a variable ratio of BC to hematological malignancies [122, 124, 125]. Given the extensive range of studies on this issue, we present only the most recent and significant meta-analyses, evaluating ACEIs and ARBs use in AIC see Table 2. Furthermore, we discuss guidelines and recommendations published by international cardiological and oncological societies, which are informed by these studies. Finally, we discuss the latest reports on two specific groups of drugs belonging to RAAS inhibitors, ARNI and MRA, and their potential use in the prevention of AIC.

**Table 2:** Summary of meta-analyses evaluating cardiovascular toxicity (CVT) prevention strategies associated with anthracyclines and/or trastuzumab oncological therapies

Metaanalysis	Studies included	Number of patients	Cancer type	Chemotherapy type	Cardiovascular Protection Strategy	Findings			
						Conclusions	OR/RR	95% CI	p-value
Kalam et al. (2013) [121]	12 RCTs and 2 observational studies	2015	BC, hematological malignancy	Anthracycline, trastuzumab and/or tamoxifen	ARBs	Reduced risk of development of HF, a drop in ejection fraction or both	RR 0.11	0.04–0.29	p < 0.0001
Abdel et al.(2017) [124]	16 RCTs	1918	BC, hematological malignancy	Anthracyclines	ACEIs or ARBs	Reduced risk of development of HF	OR 0.18	0.05–0.55	-
Avila et al.(2023) [125]	17 RCTs	1530	BC, hematological malignancy	Anthracycline and/or trastuzumab	RAASi and BBs	Smaller reduction in LVEF	MWD 4.42	2.3—6.6	p < 0.001
						Reduced risk of development of HF	RR 0.45	0.3–0.7	No data available
Lewinter et al. (2021) [11]	9 RCTs	1362	BC	Trastuzumab	ACEI/ARBs	Smaller reduction in LVEF	MD: 2.4	0.3–4.2	No data available
				Anthracycline		Decrease the reduction in LVEF during anthracycline treatment	MD: 1.5	-0.6–3.7	No data available
Li et al. (2023) [123]	13 RCTs	1905	BC, hematological malignancies, Ewing’s sarcoma, Wilms tumor	Anthracyclines	Enalapril (an ACEI)	Reduced risk of developing significant decline in LVEF	RR 0.05	0.00–0.20	No data available
Goulas et al. (2023) [12]	6 RCTs	1056	BC	Trastuzumab	ACEIs/ARBs	Reduction of cardiotoxicity occurrence	OR 0.92	0.54–1.56	p = 0.75

Only prevention strategies based on renin–angiotensin–aldosterone system inhibitors (angiotensin-converting enzyme (ACEI) and angiotensin receptor type 1 blockers (ARB)) use were included. *BC* breast cancer, *CI* confidence interval, *HF* heart failure, *LVEF* left ventricle efferent fraction, *MD* mean difference, *MWD* weighted mean difference, *OR* odds ratio, *RAASi* renin–angiotensin–aldosterone system inhibitors, *RCT* randomized controlled trials, *RR* risk ratio

First, we will focus on studies demonstrating the impact of RAASi on CVT risk. In 2013 Kalam et al. complied data from 14 published articles (n = 2015, including pediatric and adult patients), encompassing 12 RCTs and two observational studies. They reported a reduced risk of cardiac events (development of HF, a drop in ejection fraction (EF) or both) during or after chemotherapy with anthracycline and/or trastuzumab (mainly anthracycline) when using angiotensin antagonists (RR = 0.11 [95% CI 0.04–0.29], p < 0.0001) as well as BBs (RR = 0.31 [95% CI 0.16–0.63], p = 0.001) [121]. These results were quite consistent with the analysis performed by Yun et al. in 2015, which investigated early-onset AIC in the adult population only, receiving anthracyclines without trastuzumab. They showed an association of BBs and/or angiotensin antagonists’ (ACEIs and ARBs) treatment with a higher post-chemotherapy LVEF of 64.03% compared with 57.48% for control treatment. The mean difference estimate (95% CI) was 6.06% (0.54 to 11.58), p = 0.03, with significant heterogeneity, I(2) (95% CI) = 96% (82.7 to 109.3). In an exploratory subgroup analysis, the benefit of experimental agents on LVEF preservation was prominent in patients treated with higher cumulative dose of anthracyclines, but not in the lower dose group. Another subgroup analysis demonstrated an association of earlier administration of prophylactic drugs (before or concomitant to chemotherapy) with better LVEF preservation and fewer cardiac events [126]. In Abdel et al.’s Bayesian network meta-analysis, published in 2017, 16 trials (n = 1918 adult patients, 66% BC, 19% hematological malignancies; only 17% male) were included. The study was planned to use hierarchal outcome definitions in the following order of priority: (1) composite of HF or decline in LVEF, (2) decline in EF, or (3) HF. The study demonstrated angiotensin antagonists to have an 84% probability of being most effective in a sensitivity analysis excluding one outlying study (OR 0.06 [95% CI 0.01– 0.24]). When the outcome was restricted to HF angiotensin antagonists were associated with OR of 0.18 (95% CI 0.05–0.55) [124].

In 2018 Gujral et al. showed some contrary results. The study identified 8 studies (n = 1048 adult patients with various cancer types treated with anthracyclines ± trastuzumab), investigating (1) the difference in the change in LVEF or (2) the risk of new HF diagnosis between patients receiving ACE inhibitor/beta-antagonist or control. Patients treated with ACEI had no difference in both outcome categories whilst BB use was associated with a significantly smaller drop in LVEF compared to control (MWD -3.28 (95% CI: -6.1 to -0.51), p = 0.02) but not in patients who received anthracycline chemotherapy alone (MWD—3.05 (95% CI -7.22 to 1.12), p = 0.15). There was a significant reduction in new HF diagnosis in those receiving BBs compared to those not (OR 0.33 (95% CI: 0.14–0.80), p = 0.01) [125]. In 2023, Avila et al. evaluated 17 RCTs in adults receiving anthracycline chemotherapy. The study showed that the use of RAASi or BBs to prevent AIC was associated with less pronounced reduction in LVEF (weighted mean difference (MWD) 4.42 [95% CI 2.3 to 6.6]), higher final LVEF (p < 0.001), and lower incidence of HF (risk ratio 0.45 [95% confidence interval 0.3 to 0.7]). No changes in mortality were observed (p = 0.3) [122].

Lewinter et al. in 2021 as well as Gao et al. in 2023 investigated the benefits of preventive strategies in BC women patients. Lewinter et al. included 9 RCTs (n = 1362) and showed that BB and ACEI/ARBs decrease the reduction in LVEF during trastuzumab and anthracycline treatments [MD: 2.4; 95% CI: 0.3–4.2 and MD: 1.5; 95% CI: -0.6 to 3.7]. Compared with placebo, LVEF was significantly higher in patients assigned to BB or ACEI/ARB on trastuzumab (MD: 2.3; 95% CI: 0.0–4.6) but not on anthracyclines (MD: 1.9; 95% CI: -0.5 to 4.2) [11]. Gao et al. gathered 15 RCTs (n = 1977) and confirmed that ACEI/ARBs as well as BB, when compared to placebo, can protect BC patients from cardiotoxicity induced by trastuzumab and anthracycline-containing regimens, suggesting that both are helpful [127].

Additionally, in 2023, Li et al. in Bayesian network meta-analysis performed a head-to-head comparison of RAAS blockade regimens for long-term chemotherapy-related cardiac dysfunction. The study evaluated 13 RCTs (n = 1905 patients) and showed that only enalapril (RR 0.05, 95% CI 0.00–0.20) was associated with reduced risk of developing significant decline in LVEF relative to placebo. Such efficacy in protection was only in patients treated with anthracyclines and not with trastuzumab. None of RAASi protected against toxicities related to treatment with both anthracycline and trastuzumab. Moreover, the RAAS blockade therapy did not conclusively influence other markers of cardiac function, including left ventricular diastolic function and cardiac biomarkers [123].

Finally, in 2023, Goulas et al. have taken a closer look at the patients who received trastuzumab only as primary or adjuvant therapy. They analyzed 6 RCTs (n = 1056, predominantly non-metastatic, HER-2 positive BC) and found no reduction of cardiotoxicity occurrence after the use of ACEIs/ARBs and BB, compared to controls (OR) = 0.92, 95% CI 0.54–1.56, p = 0.75). However, the beneficial effect in maintaining LVEF as well as decreased number of trastuzumab therapy interruptions has been noticed [12].

Data regarding the impact of ACEIs/ARBs on OS, PFS or malignancy response rate in patients undergoing anthracycline ± anti-HER2 chemotherapy are relatively modest. In a retrospective study (n = 1449) in 2013, Chae et al. found no differences in pathologic complete response (pCR) or OS between ACEI/ARB users and non-users among BC patients receiving neoadjuvant chemotherapy [120]. In 2018, Wittayanukorn et al. utilized Surveillance, Epidemiology and End-Results-Medicare-linked (SEER) database to identify 6543 women with BC who received anthracyclines or trastuzumab. The adjusted hazard ratio for all-cause mortality for the ACEI/BB exposed group was 0.79 (95% confidence interval, 0.70–0.90) compared with the non-exposed group. A decrease in all-cause mortality was also associated with ACEIs/BBs use < / = 6 months after the initiation of trastuzumab/anthracyclines chemotherapy and a duration of ACEIs/BBs use > / = 6 months [33].

Prospective clinical trials, providing data with a higher level of evidence, showed promising results, although they had serious limitations mainly due to challenging comparability of control groups. In an RCT (n = 90), Bosch et al. demonstrated that patients with hematological malignancies treated with combined enalapril and carvedilol had a decreased incidence of the combined event of death or HF (6.7% vs. 22%, p = 0.036) and of death, HF, or a final LVEF < 45% (6.7% vs. 24.4%, p = 0.02), compared to the control group [34]. Długosz-Danecka et al. in another small RCT (n = 35) showed that patients diagnosed with NHL receiving a primary cardioprotection (ramipril and/or bisoprolol) did not experience cardiovascular deaths, in contrast to the retrospective group where cardiovascular mortality was 12.5% at 3 years (p < 0.05) [128]. Moreover, the study demonstrated a trend towards increased response rates (complete response 82 vs. 67%; p not significant) and prolonged survival (projected 5-year OS 74 vs. 60%; p < 0.05) for patients treated with primary cardioprotection [128].

Some of the aforementioned meta-analyses investigating cardioprotection in the context of anthracycline therapy refer to the impact of anthracyclines on mortality and oncological response. Abdel et al. found no association between preventive RAASi use and malignancy response rate or risk of all-cause death (the risk was similar to control with a median OR of 0.74 (95% CrI 0.16–3.51) for angiotensin antagonists and 0.42 (95% CrI 0.08–1.85) for BBs [124]. Avila et al. also observed no changes in mortality in anthracycline-treated patients receiving RAASis or BBs (p = 0.3) [122].

As indicated above, the use of preventive RAASi with anthracycline ± anti-HER2 chemotherapy remains questionable. Studies exhibit heterogeneity, employing various methodologies and definitions for cardiotoxicity, malignancy response, and survival. Some studies lack randomization or face challenges in ensuring comparability of control groups. Larger RCTs with long-term follow-up are needed to assess outcomes reliably. These RCTs should be tailored for specific cancer types and particular populations, including children [13, 121]. Additionally, more RCTs involving patients’ co-morbidities are needed to identify high-risk patient groups for whom preventive treatment may be beneficial. Patients diagnosed with hypertension may derive greater benefit from ACEIs/ARBs preventive treatment whilst those with a prior myocardial infarction may benefit more from BBs [12].

Several guidelines informed by the results of conducted clinical trials gradually assumed an attitude toward the preventive use of RAASi during anthracycline ± anti-HER2 chemotherapy. Initially, in American Society of Clinical Oncology Clinical Practice Guideline 2017 RAASi-based preventive strategy was named as an ongoing area of active investigation but was not included among recommendations [129]. Further, European Society of Medical Oncology (ESMO) Guidelines 2020 in patients with a normal LVEF and cardiovascular risk factors who are scheduled to undergo anticancer therapy with known cardiotoxic agents, particularly those exposed to multiple cardiotoxic agents, prophylactic use of ACEIs or ARBs (if intolerant to ACEIs) and/or selected BBs may be considered to reduce the development of cardiotoxicity [II, B] [130]. Ultimately, European Society of Cardiology (ESC) Guidelines 2022 suggest that ACEI or ARB and BB recommended for HF should be considered for primary prevention in high- and very high-risk patients receiving anthracyclines and/or anti-HER2 therapies [class of recommendation IIa, level of evidence B] [7].

Additionally, valsartan/sacubitril, an ARNI, recently has gained attention as a preventive strategy for AIC [92]. At least seven preclinical studies evaluated the benefits of ARNI use in this context, as extensively reviewed by Sobiborowicz et al. [92]. However, human studies assessing the efficacy of ARNIs are scarce. In a prospective study (n = 635), Gregorietti et al. demonstrated that ARNIs are safe and effective in patients with CTRCD who were previously exposed to chemotherapy for BC (mainly anthracyclines and anti–HER2 treatment) [131]. Currently, there is no human-derived data on ARNIs for primary prevention of AIC. Nevertheless, an ongoing multicenter, randomized, placebo-controlled, double-blind phase-2 clinical trial, PRADAII (NCT03760588), is evaluating the efficacy of ARNI during anthracycline-containing (neo-)adjuvant chemotherapy for BC in the primary prevention of CTRCD [132].

MRAs have also been evaluated in the setting of AIC [98]. In 2018, Lother et al., using a murine model of AIC, showed that eplerenone prevented left ventricular dysfunction induced by doxorubicin, with the beneficial effect attributed to inhibition of the mineralocorticoid receptor in cardiac myocytes [97]. In another murine model focused on the primary prevention of acute and chronic AIC, Hullin et al., compared the effects of eplenerone and enalapril on cardiotoxicity markers. They found that eplenerone administration, unlike enalapril, did not protect cardiac muscle and was associated with increased plasma aldosterone levels [98].

To date, there are only two modest clinical trials investigating MRA use in AIC. Akpek et al., in a single-center RCT (n = 83) showed that spironolactone (25 mg/kg) administrated concurrently with anthracyclines, protects both myocardial systolic and diastolic functions [133]. In another single-center RCT (n = 44), named ELEVATE, Davis et al., found that the concomitant administration of eplerenone for 6 months was not associated with significant differences in systolic or diastolic function compared with placebo in patients with early or locally advanced breast cancer treated with anthracycline-based chemotherapy [134]. Despite these findings, larger multicenter RCTs are needed to reliably assess MRA efficacy in the prevention of AIC.

## RAASi in VSPI cancer therapy: navigating cardioprotection, survival, and treatment response

Angiogenesis is one of the critical factors conditioning cancer progression, including local invasion and metastasis formation. For over fifty years [135] the use of angiogenesis inhibitors as therapeutic strategy has been developing and currently VEGF and its signaling pathway inhibitors (VSPI) are key components in therapy of various cancers, particularly in renal cell cancer (RCC) and colorectal cancer (CRC) therapy [136–138]. The commonly used members of the group are bevacizumab, sunitinib and sorafenib, however a few new-generation drugs have been introduced in recent years [139].

The risk of CVT related to VSPI therapy has been reported and has become significant complication in patients receiving the therapy [140]. Constitutive VEGF signaling is important in cardiovascular physiology and its interruption by antiangiogenic therapies can induce endothelial dysfunction, vasoconstriction, and microvascular rarefaction. Hence, hypertension, thromboembolic events and cardiotoxicity are the most frequent complications of such therapy [16, 140].

There is a crosstalk between VEGF and classical RAAS signaling. VEGF has a compensatory role when RAAS is overactivated, hence VSPI use may cause the imbalance between the pathways and the advantage of RAAS [140, 141]. Increased classical RAAS components level plays a specific role in the development of toxicity and progression of cancer. A higher activity of ACE/Ang-II/AT-1R axis has been found to be associated with a better response to bevacizumab in patients diagnosed with BC and CRC [142]. Moreover, in patients diagnosed with metastatic CRC (mCRC), low ACE levels were significantly associated with poor OS, supporting that the RAAS components can be potential predictive markers of response to anti-angiogenic drugs [142, 143]. It is consistent with the finding that hypertension correlates with better OS and possibly PFS for metastatic RCC (mRCC) patients treated with VSPIs [144, 145]. Although these correlations, RAASi use, concomitant to oncological treatment based on VSPI use, has been shown effective in numerous studies [41, 144–146].

The idea of prophylactic use of RAASi at the onset of antiangiogenic treatment is supported by some preclinical in vivo studies. In chronic murine model, aliskiren, perindopril and valsartan prevented hypertension, adverse cardiovascular remodeling, and cardiac dysfunction [41]. On the other hand, in rodent models, captopril, an ACEI, did not prevent blood pressure rise but was suitable to prevent proteinuria, suggesting its nephroprotective efficacy [105]. These data are relatively limited, as the observation period was only 8 days, and captopril is not a dedicated drug for long-term hypertensive therapy. Another study also showed inferiority of captopril in comparison to nifedipine, a calcium channel blocker, in hypertension management [147]. Preclinical data also showed synergistic effect of bevacizumab and lisinopril, an ACEI, in the treatment of CRC. In murine model with HCT116 colon cancer cell xenografts they synergistically inhibited subcutaneous tumor growth and enhanced the concentration of 5-fluorouracil (5-Fu) in tumor tissues [148]. Additionally, lisinopril did not impede the vascular normalization effect caused by bevacizumab, but also inhibited collagen and hyaluronic acid (HA) deposition and significantly downregulated the expression of TGF-beta1 and downstream SMAD signaling components which were enhanced by bevacizumab, ultimately remodeling primary extracellular matrix components [148]. Some research recommends caution in using RAASi as they can impact various aspects related to the tumor microenvironment and thus act in favor of the tumor. Among such mechanisms are local immunosuppressive effects, accumulation of bradykinin peptides, substance P, and Ac-SDKP, as well as the counteraction of the antiangiogenic effect of VSPIs and decrease in their clinical activity, leading even to relapse in patients with long-term remission [149, 150].

The prophylactic use of RAASi has been studied in several clinical studies of different design and level of evidence see Table 3. In 2011 Keizman et al. in retrospective examination showed that RAAS blockade can improve the outcome of sunitinib treatment in mRCC. However, the study did not specify whether it was due to the impact on cardiovascular system [146]. Further in 2015 Izzedine et al. in a retrospective study tried to examine the association between hypertension, RAASi use and survival outcomes in patients with mRCC treated with sunitinib. The study showed that hypertensive patients were significantly associated with longer OS (p = 0.05) and marginally with longer PFS (p = 0.06) then non-hypertensive patients and RAASi intake was significantly associated with better OS (p < 0.001) and PFS (p = 0.009). There was no difference between patients who receive RAASi before starting sunitinib therapy and those who received RAASi during sunitinib therapy [144]. The results were partially confirmed in Penttila et al. study, in 2017, among patients with mRCC treated with sunitinib or pazopanib. The study demonstrated OS and PFS benefit for RAASi users with sunitnib/pazopanib-induced hypertension whilst the impact on patients with no treatment-induced hypertension was not significant [145]. A larger study (n = 3,511) by Hamnvik et al. of mRCC patients treated with various VSPIs, including sunitinib, sorafenib, axitinib, bevacizumab, found that RAASi users had improved OS compared to nonusers, and that this association was not as prominent for patients treated with non-VEGF-targeted agents [35]. In contrast McKay et al., in a similar but smaller study (n = 1120) of various VSPIs and across patients with various cancer types, did not find significant association between RAASi use and OS. None of these studies reported analyses to assess whether the association was consistent between different VSPI or different RAASi (ACEIs vs. ARBs) [151]. Sorich et al. in pooled analysis of two RCTs (n = 1545) found no significant association between RAASi use at baseline and improved OS in patients using pazopanib or sunitinib [152]. Exploratory analyses suggested that the association may potentially be stronger for patients treated with sunitinib rather than pazopanib and with use of an ARB rather than an ACEI. The latter may be explained by the difference in the mechanism of action of these drugs: ARBs block AT-1R leaving AT-2R free to bind Ang-II, which is known to mediate anti-angiogenic effect [153].

**Table 3:** Summary of retrospective studies evaluating benefits from RAASi use concomitantly to VSPI oncological therapies in patients with metastatic renal cell carcinoma (mRCC)

Study	Number of patients	Chemotherapy type	Findings			
			Conclusions	OR/RR	95% CI	p-value
Keizman et al. [146]	127	Sunitinib	Increased incidence of sunitinib induced hypertension in RAASi users	-	-	0.82
Izzedine et al. [144]	213	Sunitinib	Longer OS and PFS in RAASi users	-	-	< 0.0001
Penttila et al. [145]	129	Sunitinib or pazopanib and TKI	Improved OS in RAASi users	-	-	< 0.001
			Improved PFS in RAASi users	-	-	< 0.004
Hamnvik et al [35]	3511	Sunitinib, sorafenib, axitinib or bevacizumab	No differences in outcomes in RAASi users	0.89	0.73–1.07	0.2018
McKay et al. [151]	1120	Sunitinib, sorafenib, axitinib, temsirolimus, bevacizumab, interferone alpha	Improved OS in RAASi users	0.711	0.625–0.809	0.0105
Sorich et al. [152]	1545	Sunitinib or pazopanib	Improved OS in RAASi users treated with sunitinib	-	0.55–0.98	0.03

Only prevention strategies based on renin–angiotensin–aldosterone system inhibitors (RAASi) use were included. *OR* odds ratio, *PFS* progression free survival, *RCT* randomized controlled trials, *RR* risk ratio, *TKI* tyrosine kinase inhibitors

Finally, the exploratory analysis showed that the use of calcium channel blockers or BBs at baseline is associated with improved OS, which may suggest that baseline hypertensive treatment is beneficial even without RAAS blockade or that there is a direct effect of calcium channel blockers on cancer [154]. In 2023, van Dorst et al. shed light on the topic in a clinical cohort study, identifying the following determinants of VSPI-induced blood pressure rise: (1) the type of VSPI (pazopanib was identified as the most prohypertensive one), (2) baseline BP (normotensive patients at baseline were more likely to develop VSPI-induced hypertension), (3) baseline glomerular filtration rate (GFR) < 60 mL/min per 1.73 m2. Both RAASi and calcium channel blockers were shown effective in antihypertensive treatment. The impact of blood pressure rise on OS for the first time has been shown to be cancer type dependent [155].

ESC Guidelines 2022 recommend considering the use of ACEI or ARB as well as BB for primary prevention in high- and very high-risk patients receiving targeted cancer therapies that may cause HF, including VSPIs [class of recommendation IIa, level of evidence C] [7].

## RAASi in immune checkpoint inhibitors cancer therapy: navigating cardioprotection, survival, and treatment response

The recognition of the immune escape response of tumor cells through the inactivation of cellular immunity has been a breakthrough in the treatment of numerous solid and hematological malignancies [156]. A group of novel drugs, ICIs, targets crucial immunosuppressive checkpoints to unblock the anti-tumor immune response. The clinical use of these drugs has been shown to extend OS of cancer patients [157]. Commonly used drugs from the group are: (1) anti-cytotoxic T lymphocyte-associated protein-4 antibodies (anti-CTLA-4) such as Ipilimumab, (2) anti-programmed cell death protein-1 antibodies (anti-PD-1) such as Pembrolizumab and Nivolumab, (3) anti-PD-1 ligand antibodies (anti-PD-L1) such as Atezolizumab [158, 159].

Shortly after the introduction of these drugs CVTs have been reported. Myocarditis, pericarditis, arrhytmias, myocardial infarction and left ventricular dysfunction without evidence of myocarditis are the most common clinical presentations [14]. The risk of major adverse cardiac events in patients treated with ICIs is increased four-fold [160] while the most alarming is rare (1.14% according to Mahmood et al. retrospective study from 8 clinical centers[161]) but extremely severe myocarditis (with fatality rate around 35–50%) [162]. Patients treated with anti-CTLA-4 agents or with a combination of ICIs have a higher incidence of cardiotoxicity and mortality than those treated with single PD-1 inhibitors [14]. The underlying mechanism of ICI-mediated toxicity is partially recognized and may be due to the expression of PD-L1 on the surface of normal cardiomyocytes. Such surface PD-L1 molecules physiologically bind to PD-1 on T lymphocytes to prevent their immune response, but ICI use disrupts such suppressive effects and leads to the development of ICI-induced toxicity [163].

Data on the influence of ICI treatment on RAAS components expression and activity are scarce, however, some retrospective analyses have been performed to assess the impact of RAASi use on OS, PFS as well as adverse events rate in oncological patients treated with ICI see Table 4.

**Table 4:** Summary of retrospective studies evaluating benefits from RAASi use concomitantly to immune check-point inhibitors (ICIs)-based oncological therapies

Study	Number of patients	Cancer type	Immune therapy type	Findings			
				Conclusions	HR	95% CI	*p*-value
Kichenadasse et al. [164]	2539	Lung, renal and urothelial cancer	atezolizumab	No difference in PFS and OS between RAASi users and non-users	-	-	> 0.05
Drobni et al. [36]	10333	Breast, gastrointestinal, genitourinary, gynaecological, head and neck, hematological, melanoma, neurological, sarcoma, thoracic	Various ICIs	Better OS in RAASi users	0.92	0.85–0.99	0.032
Nuzzo et al. [165]	229	mRCC	Various ICIs	Improved OS and TTF in RAASi users	0.59 0.60	0.37–0.95 0.43–0.85	0.03 0.0034
Chiang et al. [37]	734	Bone, breast, gastrointestinal, gynecological, head and neck, hepatobiliary, lung, pancreatic, renal, skin	Various ICIs	Decreased risk of mortality in RAASi users	0.58	0.44–0.76	< 0.001
				Decreased risk of disease progression in RAASi users	0.62	0.50–0.77	< 0.001
				Greater clinical benefit rate in RAASi users	-	-	0.006

Only prevention strategies based on renin–angiotensin–aldosterone system inhibitors (RAASi) use were included. *HR* hazard ratio, *PFS* progression free survival, *RCT* randomized controlled trials, *RR* risk ratio

In 2021 Kichenadasse et al. performed a post hoc analysis of individual patient data from 7 clinical trials (n = 3695; 2539 were treated with atezolizumab and the rest with chemotherapy) of solid cancer treatment, including lung, renal and urothelial cancers, 24% of patients were on RAASi at trial commencement. The study did not reveal any significant difference in OS, PFS or immune adverse events between RAASi users and non-users in the atelizumab-treated cohort [164] Other classes of antihypertensives were also not associated with survival [164].

In another large retrospective study (n = 10333) Drobni et al. showed that patients with hypertension who were concomitantly taking a RAASi during ICI therapy had better OS and that benefit was primarily noted among patients with gastrointestinal and genitourinary cancers [36]. Nuzzo et al. in multicenter retrospective study (n = 229) showed that concurrent RAASi administration was associated with improved OS and time-to-treatment failure (TTF) in mRCC patients [165]. Chiang et al. in retrospective cohort study (n = 734) showed that RAASi use, particularly ARB, was associated with improved clinical outcomes across a broad range of cancer patients receiving ICI [37]. RAASi use was not associated with increased risk of adverse events [37]. However, the use of RAASi before immune checkpoint blockade was not associated with improved survival [37].

ICIs are novel drugs and data about their toxicity and preventive strategies is limited. Further research, including randomized studies with clinically relevant endpoints, is needed to show high-risk patient groups, in which cardiovascular protection, including RAASi, may be beneficial. Until now, clinical guidelines don’t mention RAASi use concomitant to ICIs chemotherapy [7, 130].

The combination therapy of VSPI and ICI is utilized in patients with various solid malignancies, including mRCC [166], HCC [167] and non-small cell lung cancer (NSCLC) [168, 169], enhancing treatment efficacy. It is highly probable that soon it may become a significant therapeutic strategy for many other malignancies eg. cervical metastatic cancer [170] or recurrent ovarian cancer [171]. The combinatory approach provides synergistic anti-tumor effect, but simultaneously it is accompanied by the increase in cardiovascular risk, as some specific toxicities of both groups overlap [32]. According to a real-world pharmacovigilance analysis based on the FDA Adverse Event Reporting System (FAERS) database from 2014 to 2022, combination therapy has increased risk of cardiovascular adverse effects than ICIs alone and it is primarily due to an increase in embolic and thrombotic events [172]. However, the study showed that the risk of noninfectious myocarditis/pericarditis is lower. Additionally, combination therapy is associated with lower frequency of death and life-threatening adverse effects [172]. A meta-analysis of RCTs, comparing CVTs of combination therapy with VSPI and ICI versus VSPI alone also suggests increased CV risk [173]. The impact on RAAS imbalance during the combination therapy has not yet been studied, but regarding the data on dysregulation of RAAS axes during VSPI therapy alone, it may be speculated that changes occur, providing rationale for RAASi use [41, 142, 148]. Future RCTs with clinically relevant endpoints are needed to assess whether such preventive RAASi treatment could improve OS of the patients and lower the risk of CVTs.

## Conclusions and future perspectives

Several studies have assessed the use of RAASi as a cardiovascular protection strategy and/or adjunct therapy in patients undergoing oncological treatment. This concept is strongly supported by evidence indicating dysregulation of RAAS components in both the tumor microenvironment and vital organs affected by anticancer drug toxicities. The imbalance between the two main RAAS axes, primarily shifting towards the classical axis, contributes to malignant transformation of the tumor, including angiogenesis, prosurvival signaling and invasion [2] as well as maladaptive changes in intrinsic organs, including oxidative stress, fibrosis, inflammation [40]. These changes are anticipated to impact cancer patient OS and treatment effectiveness, with RAASi altering RAAS component levels and activities to promote other protective axis, potentially yielding beneficial effects.

Our review focused on concurrent RAASi use with four distinct drug classes – anthracyclines, anti-HER2 therapies, VSPIs and ICIs—widely utilized in oncological treatment, affecting cardiovascular system and triggering toxic changes and dysfunctions. We synthesized evidence from studies of varying levels of evidence to evaluate the benefits and risks associated with preventive RAASi use.

Overall, reviewed studies present inconsistent results regarding the effectiveness of preventive RAASi use in the general oncological patient population. However, targeting specific cancer types and stages, along with identifying appropriate high-risk groups, appears more favorable for RAASi use. An example of such application could be the concurrent use of RAASi with VSPIs in mRCC therapy, or together with anthracyclines and trastuzumab therapy in mBC treatment. Other factors influencing the effectiveness of such therapy may include type of RAASi and the timing and duration of its administration [123].

Considering the advancements in clinical research utilizing MasR agonists and robust preclinical evidence indicating their cardioprotective and anticancer effects, a new therapeutic opportunity may emerge soon for oncology patients with cardiovascular complications [174]. Novel drugs, when used concomitantly with conventional RAASi, may enhance their efficacy and lead to prolonged survival in these patients. Nevertheless, at present, further large-scale RCTs are needed to substantiate these findings.

## Key References

- Sobczuk, P., et al., *Anthracycline-induced cardiotoxicity and renin–angiotensin–aldosterone system-from molecular mechanisms to therapeutic applications.* Heart Fail Rev, 2022. **27**(1): p. 295–319. 10.1007/s10741-020-9977-1.
  - This reference is of major importance as it explains how anthracyclines disrupt RAAS and lead to CVT.
- Morelli, M.B., et al., *Cardiotoxicity of Anticancer Drugs: Molecular Mechanisms and Strategies for Cardioprotection.* Front Cardiovasc Med, 2022. **9**: p. 847012. 10.3389/fcvm.2022.847012.
  - This reference is of importance because it explains mechanisms of anticancer drugs in detail.
- Shin, K., et al., *Angiotensin-converting enzyme inhibitors or angiotensin receptor blockers and cancer risk: an updated meta-analysis of observational studies.* Ther Adv Drug Saf, 2022. **13**: p. 20420986221129335. 10.1177/20420986221129335.
  - This reference is of major importance as it is a recent meta-analysis explaining how RAASi may be used in anticancer treatment.
- Avila, M.S., et al., *Renin-angiotensin System Antagonists and Beta-blockers in Prevention of Anthracycline Cardiotoxicity: a Systematic Review and Meta-analysis.* Arq Bras Cardiol, 2023. **120**(5): p. e20220298. 10.36660/abc.20220298.
  - This reference is of major importance as it explains how RAASi may alleviate adverse effects caused by anthracyclines.
- Li, X., et al., *New advances in the research of clinical treatment and novel anticancer agents in tumor angiogenesis.* Biomed Pharmacother, 2023. **163**: p. 114806. 10.1016/j.biopha.2023.114806.
  - This reference is of importance as it describes novel anticancer therapies.
- Kichenadasse, G., et al., *Effect of concomitant use of antihypertensives and immune check point inhibitors on cancer outcomes.* J Hypertens, 2021. **39**(7): p. 1274–1281. 10.1097/HJH.0000000000002799.
  - This reference is of importance as it describes the effects of RAASi and ICIs on cancer outcomes.

## Data Availability

No datasets were generated or analysed during the current study.

## Abbreviations

ACEIs: Angiotensin-converting enzyme (ACE) inhibitors
Ac-SDKP: N-acetyl-seryl-aspartyl-lysyl-proline
AIC: Anthracycline-induced cardiotoxicity
Ang-I: Angiotensin I
Ang-II: Angiotensin II
Ang-III: Angiotensin III
Ang-IV: Angiotensin IV
Ang-(1-7): Angiotensin-(1-7)
Ang-(1-9): Angiotensin-(1-9)
anti-CTLA-4: Anti-cytotoxic T lymphocyte-associated protein-4 antibodies
anti-PD-1: Anti-programmed cell death protein-1 antibodies
anti-PD-L1: Anti-PD-1 ligand antibodies
ARBs: Angiotensin receptor blockers
AT-1R: Angiotensin receptor type 1
AT-2R: Angiotensin receptor type 2
ATG: Angiotensinogen
BBs: Beta-blockers
BC: Breast cancer
CRC: Colorectal cancer
CVT: Cardiovascular toxicity
ESC: European society of cardiology
ESMO: European society of medical oncology
GFR: Glomerular filtration rate
HA: Hyaluronic acid
HER2: Human epidermal growth factor receptor 2
HF: Heart failure
HFrEF: Heart failure with reduced ejection fraction
ICIs: Immune-check-point-inhibitors
IC-OS: International cardio-oncology society
IRAP: Insulin-regulated amino peptidase
LVEF: Left ventricular ejection fraction
MasR: G-protein-coupled receptor Mas
MI: Myocardial infarction
miRNAs: MicroRNAs
MrgD: MAS1-related G-protein-coupled receptor D
MR: Mineralocorticoid receptor
MRR: Malignancy response rate
MWD: Weighted mean difference
NHL: Non-Hodgking lymphoma
NSCLC: Non-small cell lung cancer
OS: Overall survival
PFS: Progression free survival
pCR: Pathologic complete response
RAAS: Renin-angiotensin-aldosterone system
RAASi: Renin-angiotensin-aldosterone system inhibitors
RCC: Renal cell cancer
RCTs: Randomized controlled trials
SEER: Surveillance, epidemiology and end-results-medicare-linked database
TGF-beta1: Transforming growth factor beta1
TTF: Time-to-treatment failure
VEGF: Vascular endothelial growth factor
VSPI: VEGF signaling pathway inhibitors
